# Electroacupuncture attenuates neuropathic pain via suppressing BIP-IRE-1α-mediated endoplasmic reticulum stress in the anterior cingulate cortex

**DOI:** 10.1186/s40659-024-00511-3

**Published:** 2024-05-29

**Authors:** Lin-Wei Ma, Yu-Fan Liu, Hui Zhang, Chang-Jun Huang, Ang Li, Xin-Zhe Qu, Jia-Piao Lin, Yan Yang, Yong-Xing Yao

**Affiliations:** 1 Department of Anesthesia, First Affiliated Hospital, Zhejiang University School of Medicine, 79 Qingchun Road, Hangzhou, 310003 China; 2 Department of Neurobiology, Sir Run Run Shaw Hospital, Zhejiang University School of Medicine, 3 East Qingchun Road, Hangzhou, 310020 China; 3 School of Brain Science and Brain Medicine, Zhejiang University, Hangzhou, 310058 China; 4 Department of Anesthesia, First People’s Hospital of Linping District of Hangzhou City, 369 Yingbin Road, Hangzhou, 311100 China; 5 Department of Orthopedics, Graduated School, Xuzhou Medical University, 209 Tongshan Road, Xuzhou, 221004 China

**Keywords:** Endoplasmic reticulum stress, Anterior cingulate cortex, Electroacupuncture, Neuropathic pain

## Abstract

Studies have suggested that endoplasmic reticulum stress (ERS) is involved in neurological dysfunction and that electroacupuncture (EA) attenuates neuropathic pain (NP) via undefined pathways. However, the role of ERS in the anterior cingulate cortex (ACC) in NP and the effect of EA on ERS in the ACC have not yet been investigated. In this study, an NP model was established by chronic constriction injury (CCI) of the left sciatic nerve in rats, and mechanical and cold tests were used to evaluate behavioral hyperalgesia. The protein expression and distribution were evaluated using western blotting and immunofluorescence. The results showed that glucose-regulated protein 78 (BIP) and inositol-requiring enzyme 1α (IRE-1α) were co-localized in neurons in the ACC. After CCI, BIP, IRE-1α, and phosphorylation of IRE-1α were upregulated in the ACC. Intra-ACC administration of 4-PBA and Kira-6 attenuated pain hypersensitivity and downregulated phosphorylation of IRE-1α, while intraperitoneal injection of 4-PBA attenuated hyperalgesia and inhibited the activation of P38 and JNK in ACC. In contrast, ERS activation by intraperitoneal injection of tunicamycin induced behavioral hyperalgesia in naive rats. Furthermore, EA attenuated pain hypersensitivity and inhibited the CCI-induced overexpression of BIP and pIRE-1α. Taken together, these results demonstrate that EA attenuates NP by suppressing BIP- and IRE-1α-mediated ERS in the ACC. Our study presents novel evidence that ERS in the ACC is implicated in the development of NP and provides insights into the molecular mechanisms involved in the analgesic effect of EA.

**Supplementary Information:**

The online version contains supplementary material available at 10.1186/s40659-024-00511-3.

## Background

Neuropathic pain (NP) is caused by injury or disease of the somatosensory nervous system and commonly results from trauma, tumors, chemotherapy, and diabetes. NP is characterized by hyperalgesia, allodynia, and aberrant spontaneous pain [1, 2]. NP has become a serious public health issue because of its high prevalence, debilitating effects, and high social costs [3, 4]. Because the pathogenesis of NP is not yet fully understood, comprehensive treatments, such as drugs (e.g., tricyclic antidepressants, calcium channel blockers, opioids) and non-drug therapies (e.g., nerve stimulation), are commonly ineffective and often accompanied by serious side effects [5, 6]. As a component of the limbic system, the anterior cingulate cortex (ACC) is mainly involved in the emotional-affective component of pain; however, increasing evidence has recently highlighted its important role in the modulation of the sensory-discriminative component of pain, although further exploration is necessary [7, 8].

Endoplasmic reticulum stress (ERS) is a cellular protective process that is mediated by the ERS-sensing protein, pancreatic endoplasmic reticulum kinase (PERK), activating transcription factor 6 (ATF-6), inositol-requiring enzyme 1α (IRE-1α), and their downstream signaling molecules [9, 10]. Under pathological conditions, the accumulation of unfolded proteins in the endoplasmic reticulum promotes the dissociation of ERS-sensing proteins from glucose regulatory protein 78 (BIP), activating the unfolded protein response and restoring cell homeostasis [11, 12]. When hyperactivated by ERS, phosphorylated IRE-1α transitions from homodimers to high-order oligomers, inducing the activation or upregulation of numerous proinflammatory molecules, and participating in the development of multitudinous neurological diseases such as Alzheimer’s disease, Parkinson’s disease, and amyotrophic lateral sclerosis [13, 14]. The role of BIP-IRE-1α-mediated ERS in the ACC in NP remains unclear.

Acupuncture is a traditional therapeutic technique that has been used in oriental medicine for approximately 3000 years. Since the 1970s, electroacupuncture (EA) has gradually replaced manual acupuncture because of its advantages, such as better analgesic effects and objectively quantified and controlled stimulation [15, 16]. Currently, EA is widely used in stroke, Alzheimer’s disease, urinary incontinence, and other mental and physical disorders [17–19]. Recently, the analgesic effects of EA have been widely validated in basic and clinical studies [16, 20–22]. However, the mechanism underlying the antihyperalgesic effects of EA requires further elucidation.

In the present study, we aimed to investigate the following: (1) the expression of BIP and IRE-1α and the ERS mediated by BIP-IRE-1α in the ACC, (2) its role in NP, and (3) whether the mechanisms underlying the antihyperalgesic effects of EA were associated with ERS mediated by BIP-IRE-1α in the ACC.

## Results

### Cellular distribution of BIP and IRE-1α in the ACC

**Fig. 1:**
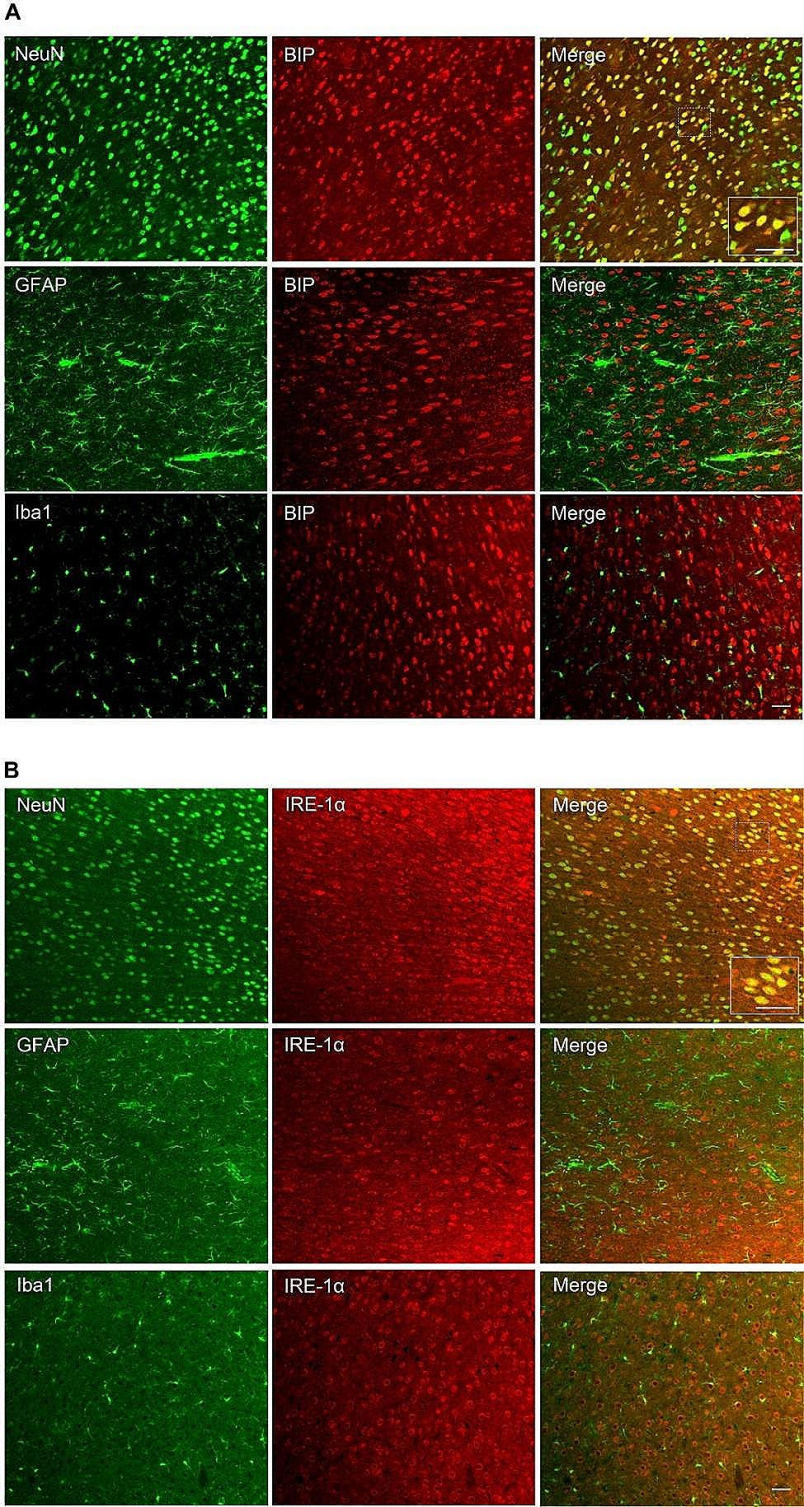
Cellular distribution of BIP and IRE-1α in the anterior cingulate cortex. Double immunofluorescence staining showed colocalization of BIP (A) and IRE-1α (B) with NeuN, but not with GFAP or Iba1. Scale bar = 50 μm. BIP: Glucose-regulated protein 78, IRE-1α: Inositol-requiring enzyme 1α, NeuN: Neuron-specific nuclear protein, GFAP: Glial fibrillary acidic protein, Iba1: Ionized calcium-binding adaptor molecule-1

We first investigated the cell types expressing BIP and IRE-1α in the ACC using immunofluorescence experiments. Double immunofluorescence staining was performed for both BIP and IRE-1α using neuron-specific nuclear protein (NeuN), glial fibrillary acidic protein (GFAP), and ionized calcium-binding adaptor molecule-1 (Iba1). The results showed that BIP and IRE-1α co-localized well with NeuN but not with GFAP or Iba1 (Fig. 1A, B).

### CCI induced behavioral hyperalgesia, upregulation of BIP and IRE-1α, and phosphorylation of IRE-1α in the ACC

**Fig. 2:**
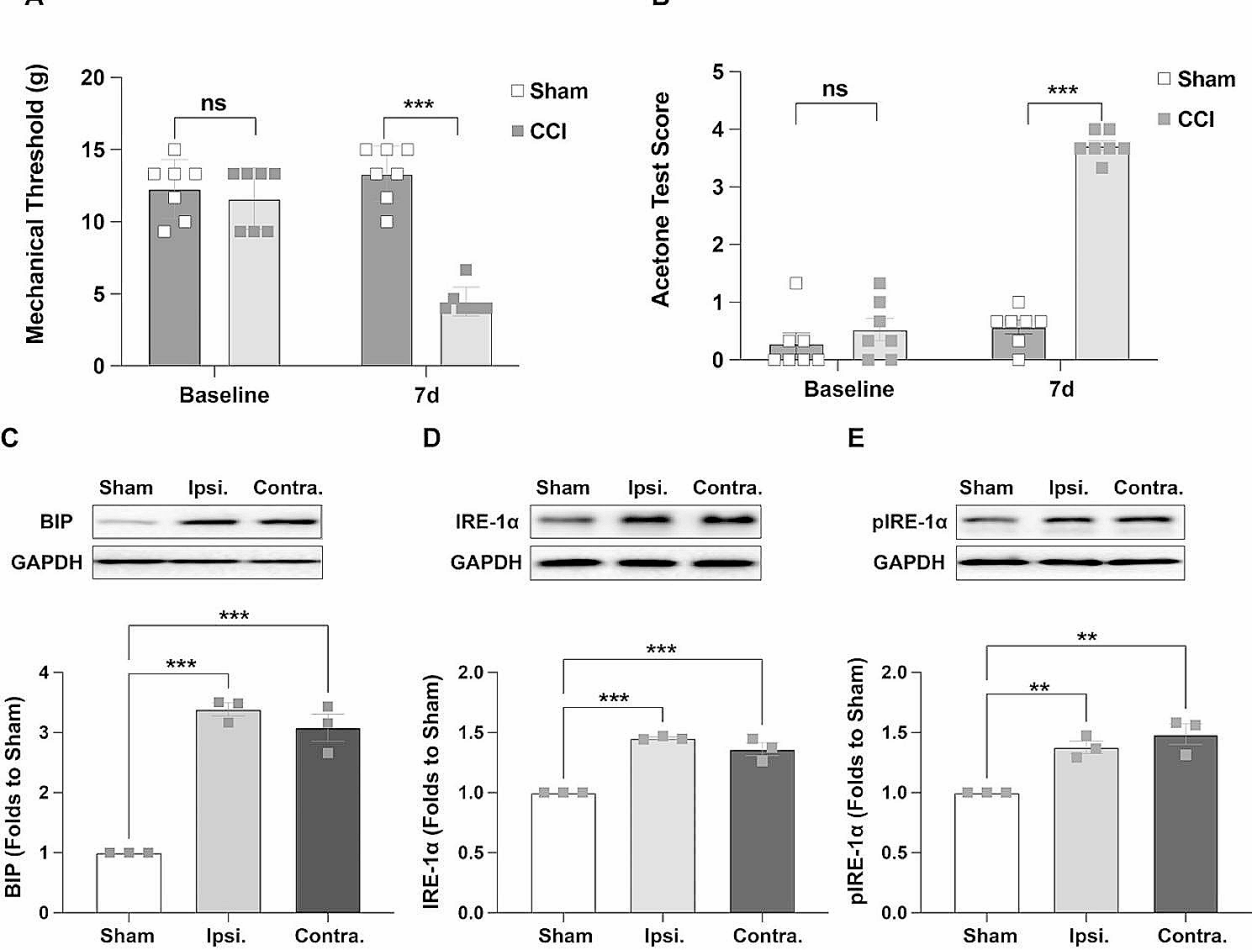
CCI induced pain hypersensitivity, upregulation of BIP and IRE-1α, and phosphorylation of IRE-1α in the ACC. (A) Compared to the sham group, the CCI group showed a significantly lower MWT on the 7th day after surgery (***p < 0.001, independent *t-*test, *n* = 7). (B) Compared to the sham group, the CCI group showed a significantly higher ATS on the 7th day after surgery (***p < 0.001, independent *t-*test, *n* = 6). (C, D, E) Western blotting showed that compared to the sham group, the expression of BIP and IRE-1α in the ACC was bilaterally increased in the CCI group (***p < 0.001, vs. sham, one-way ANOVA, *n* = 3); and the phosphorylation of IRE-1α was bilaterally increased in the CCI group (**p < 0.01, vs. sham, one-way ANOVA, *n* = 3). Error bars represent the standard error of the mean. CCI: Chronic constriction injury, BIP: Glucose-regulated protein 78, IRE-1α: Inositol-requiring enzyme 1α, ACC: Anterior cingulate cortex, MWT: Mechanical withdrawal threshold, ATS: Acetone test score

**Fig. 3:**
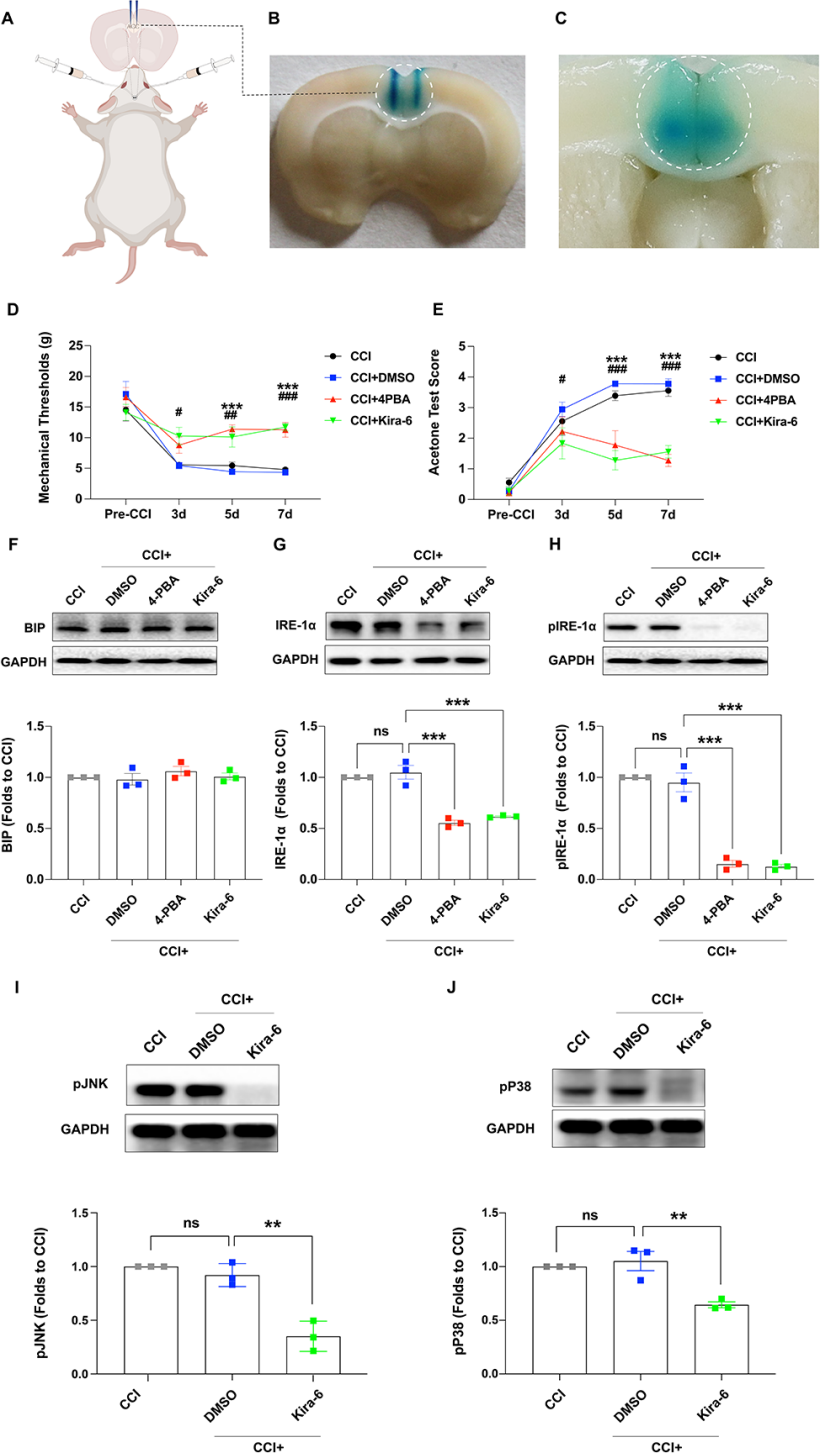
The inhibitors 4-PBA and Kira-6 reversed hyperalgesia and inhibited CCI-induced activation of IRE-1α. (A, B, C) Schematic diagram and location of ACC microinjection. (D, E) Compared to the CCI + DMSO group, the MWT and ATS were not significantly different among the four groups before CCI; the MWT was significantly higher and the ATS was significantly lower in the CCI + 4-PBA group 5 and 7 days, and 3, 5, and 7 days after CCI in the CCI + Kira-6 group (***p < 0.001, CCI + 4-PBA vs. CCI + DMSO; ^#^*p* < 0.05, ^##^*p* < 0.01, ^###^*p* < 0.001, CCI + Kira-6 vs. CCI + DMSO, two-way ANOVA, *n* = 7). (F, G, H) The results of western blotting showed that the expression of BIP was not significantly different among the four groups; however, the expression and activation of IRE-1α and pIRE-1α were significantly inhibited in the CCI + 4-PBA and CCI + Kira-6 groups compared to those in the CCI + DMSO group (***p < 0.001, one-way ANOVA, *n* = 3). (I, J) The western blotting analysis revealed a significant difference in the expression levels of pJNK and pP38 between the CCI + DMSO and CCI + Kira-6 groups (*p < 0.05, ***p < 0.001, CCI + Kira-6 vs. CCI + DMSO, one-way ANOVA, *n* = 3). Error bars represent the standard error of the mean. ACC: Anterior cingulate cortex, DMSO: Dimethyl sulfoxide, MWT: Mechanical withdrawal threshold, ATS: Acetone test score, BIP: Glucose-regulated protein 78, IRE-1α: Inositol-requiring enzyme 1α

A NP model was established by chronic constriction injury of the left sciatic nerve to explore the role of ERS in the ACC during NP. The MWT and ATS were determined before and 7 days after CCI, with no significant difference observed at baseline between the sham and CCI groups. Seven days after CCI, the MWT (Fig. 2A) was significantly lower and the ATS (Fig. 2B) was significantly higher in the CCI group than those in the sham group, demonstrating that CCI successfully induced behavioral hyperalgesia. Consistent with the behavioral hyperalgesia observed after CCI, the western blotting results showed that the expression of BIP (Fig. 2C) and IRE-1α (Fig. 2D) was bilaterally upregulated and the phosphorylation level of IRE-1α (Fig. 3E) was bilaterally increased in the CCI group on day 7 after CCI. These data demonstrated that BIP-IRE-1α mediates ERS in the ACC after CCI.

### 4-PBA and Kira-6 reversed hyperalgesia and inhibited CCI-induced IRE-1α activation

To further investigate the role of ERS in the ACC in the initiation of NP, the BIP inhibitor 4-PBA and the IRE-1α inhibitor Kira-6 were injected into the ACC from days 0 to 6 after CCI to suppress ERS in the ACC (Fig. 3A, B, C). The MWT and ATS were measured before and 3, 5, and 7 days after CCI. The behavioral test results showed that the MWT and ATS did not differ significantly among the four groups before CCI; however, the MWT (Fig. 3D) was significantly higher and the ATS (Fig. 3E) was significantly lower in the CCI + 4-PBA and CCI + Kira-6 groups than those in the CCI + DMSO group. These results indicate that CCI-induced ERS plays an important role in the initiation of NP. Meanwhile, western blotting analysis showed that the expression level of BIP (Fig. 3F) was not significantly different among the four groups, and the expression of IRE-1α (Fig. 3G) and activation of IRE-1α (Fig. 3H) were inhibited in the CCI + 4-PBA and CCI + Kira-6 groups, indicating successful suppression of ERS in the ACC. Furthermore, the results of western blot demonstrated a notable reduction in the expression levels of phosphorylated JNK (pJNK) (Fig. 3I) and phosphorylated P38 (pP38) (Fig. 3J) in the CCI + Kira-6 group, suggesting a link between the inhibition of ERS and the downregulation of P38 and JNK in the context of hyperalgesia.

### Intraperitoneal injection of 4-PBA attenuated CCI-induced hyperalgesia

**Fig. 4:**
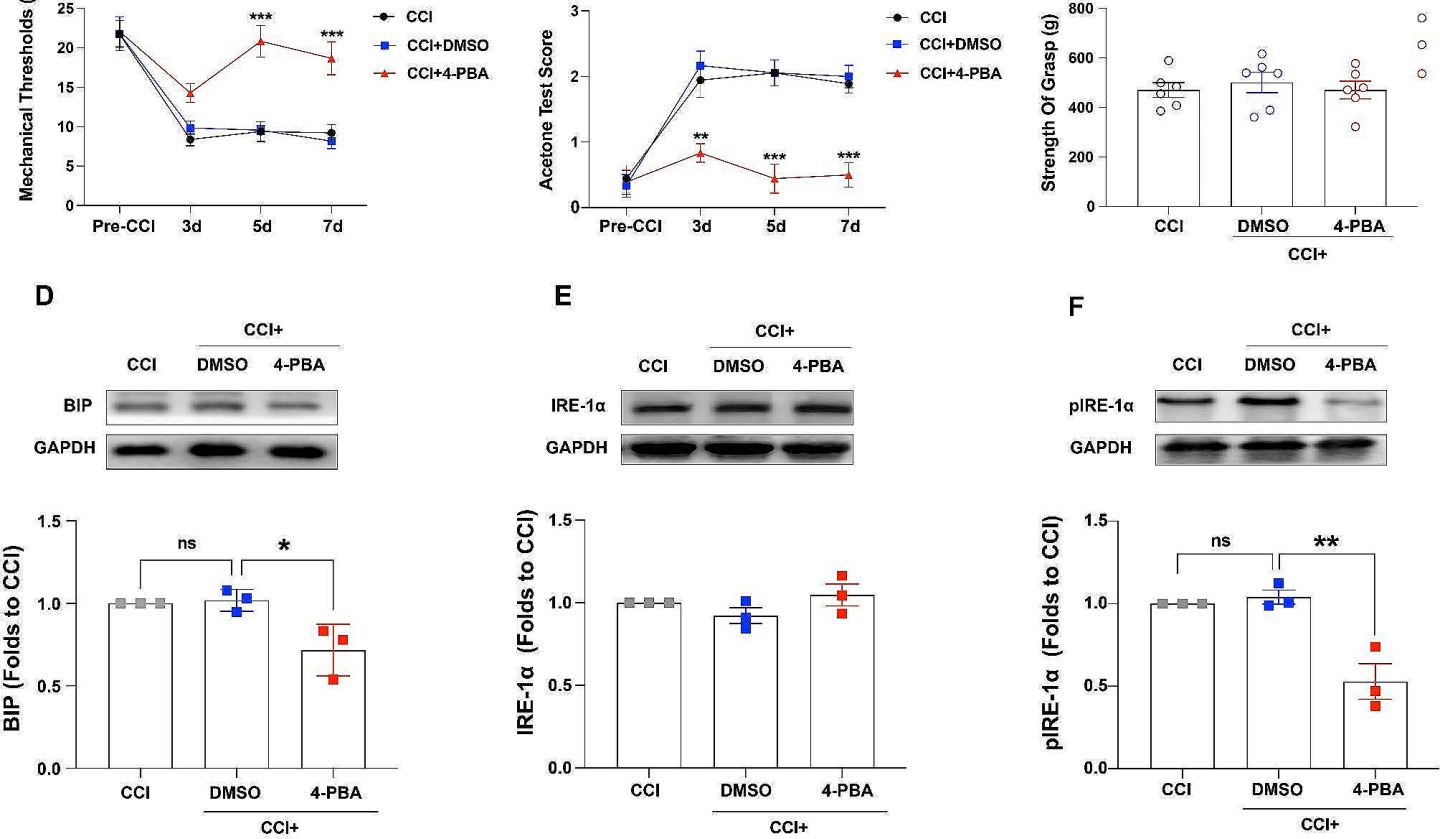
Intraperitoneal injection of 4-PBA reversed hyperalgesia induced by CCI and suppressed CCI-triggered BIP expression and IRE-1α activation. (A, B) Compared to the CCI + DMSO group, the MWT and ATS were not significantly different in the CCI + 4-PBA before CCI; the MWT was significantly higher on days 5 and 7, and the ATS was significantly lower on days 3, 5, and 7 in the CCI + 4-PBA group after CCI (**p < 0.01, ***p < 0.001, CCI + 4-PBA vs. CCI + DMSO; *n* = 6). (C) The strength of grasp of the front paws showed no significant differences among the CCI, CCI + DMSO, and CCI + 4-PBA groups (*n* = 6). (D, E, F) The expression of IRE-1α was not significantly different among the three groups, but that of BIP and pIRE-1α was significantly decreased in the CCI + 4-PBA group (*p < 0.05, **p < 0.01, one-way ANOVA, *n* = 3). Error bars represent the standard error of the mean. CCI: Chronic constriction injury, BIP: Glucose-regulated protein 78, IRE-1α: Inositol-requiring enzyme 1α, MWT: Mechanical withdrawal threshold, ATS: Acetone test score

To further explore the influence of ERS on CCI-induced hyperalgesia from a translational perspective, intraperitoneal injections of 4-PBA were administered on the day of CCI and continued once a day for the following 7 days. The MWT and ATS were measured before CCI and on days 3, 5, and 7 after CCI. The results revealed a significant increase in the MWT (Fig. 4A) and a substantial reduction in the ATS (Fig. 4B) in the CCI + 4-PBA group, with no significant impact on locomotive ability (Fig. 4C), as compared to the CCI + DMSO group. Furthermore, western blotting analysis indicated that BIP expression was inhibited in the CCI + 4-PBA group (Fig. 4D). Although the expression level of IRE-1α was not significantly different between the three groups (Fig. 4E), the activation of pIRE-1α was inhibited in the CCI + 4-PBA group (Fig. 4F). These data suggest that the inhibition of ERS might alleviate CCI-induced hyperalgesia without affecting locomotive ability, the underlying mechanisms of which warrant further investigation.

### Tunicamycin-induced hyperalgesia in naive rats

**Fig. 5:**
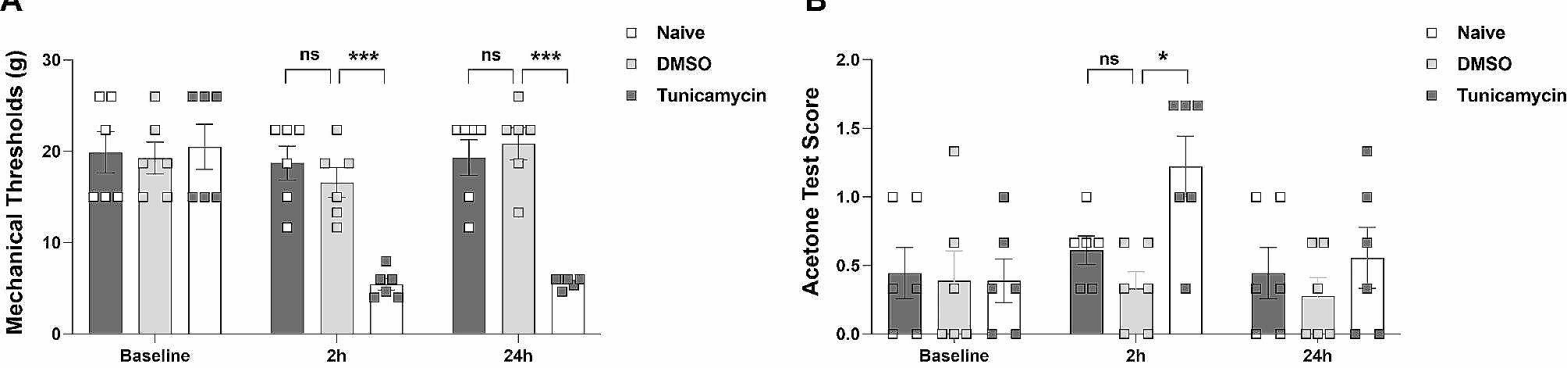
Tunicamycin injection induces hyperalgesia in naive rats. The tunicamycin group exhibited significantly lower mechanical withdrawal threshold (A, 2 and 24 h) and higher acetone test score (B, 2 h) than the DMSO group post-injection (*p < 0.05, ***p < 0.001, two-way ANOVA, *n* = 6). Error bars represent the standard error of the mean. DMSO: Dimethyl sulfoxide

To investigate whether ERS induction could lead to hypersensitivity, we next administered intraperitoneal injections of tunicamycin, a known activator of ERS, into naive rats. The MWT and ATS were assessed before and 2 and 24 h after injection. The behavioral results demonstrated that, compared to the DMSO group, the tunicamycin group exhibited a substantial decrease in MWT (at both 2 and 24 h), and a significant increase in ATS (2 h) after application (Fig. 5A, B). These findings indicate that ERS activation induces hyperalgesia in naive rats.

### EA attenuated hyperalgesia and suppressed CCI-induced ERS in ACC

**Fig. 6:**
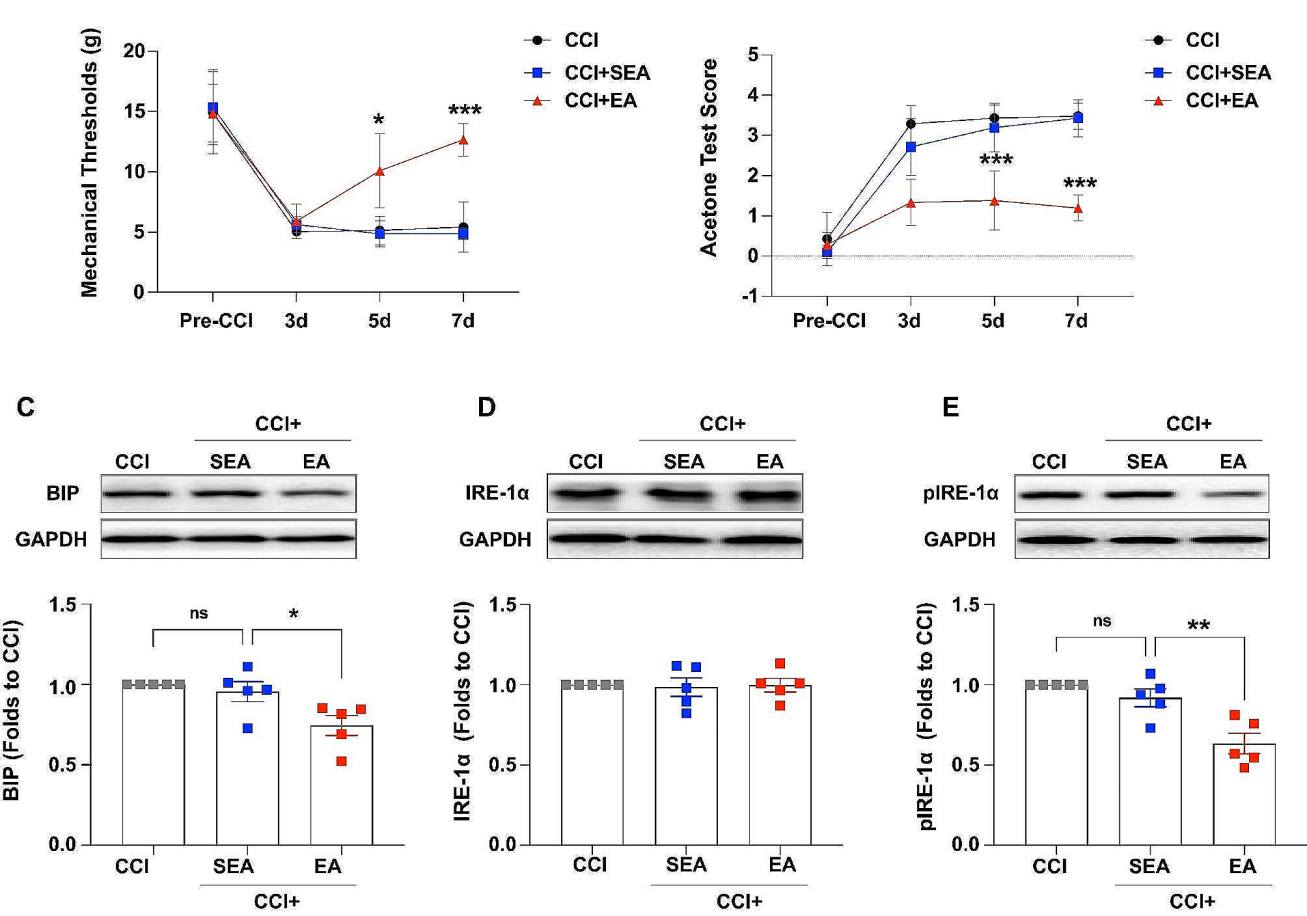
Electroacupuncture attenuated hyperalgesia and suppressed CCI-induced endoplasmic reticulum stress in the anterior cingulate cortex. (A, B) Compared to the CCI + SEA group, the MWT and ATS showed no significant differences among the three groups before and 3 days after CCI. However, the MWT was significantly higher and the ATS was significantly lower in the CCI + EA group on days 5 and 7 after CCI (**p < 0.01, ***p < 0.001, CCI + EA vs. CCI + SEA, two-way ANOVA, *n* = 7). (C) Compared to the CCI + SEA group, the expression of BIP was significantly lower in the CCI + EA group (*p < 0.05, one-way ANOVA, *n* = 5). (D, E) The expression of IRE-1α was not significantly different among the three groups, but the phosphorylation of IRE-1α was significantly decreased in the CCI + EA group (**p < 0.01, one-way ANOVA, *n* = 5). Error bars represent the standard error of the mean. CCI: Chronic constriction injury, SEA: sham EA, EA: electroacupuncture, BIP: Glucose-regulated protein 78, IRE-1α: Inositol-requiring enzyme 1α, MWT: Mechanical withdrawal threshold, ATS: Acetone test score

To further investigate the antihyperalgesic effect and mechanisms of EA, the consecutive EA procedure was applied from days 0 to 6 after CCI for 30 min/day. The MWT and ATS were measured before and on days 3, 5, and 7 after CCI. The behavioral results showed that the MWT and ATS were not significantly different among the four groups before and 3 days after CCI; however, the MWT (Fig. 6A) was significantly higher and the ATS (Fig. 6B) was significantly lower in the CCI + EA group than in the CCI + SEA group, indicating that EA inhibits the initiation of NP. Further, western blotting showed that the expression of BIP was significantly lower in the CCI + EA group than that in the CCI + SEA group (Fig. 6C). The expression of IRE-1α was not significantly different among the three groups (Fig. 6D), but the phosphorylation of IRE-1α was significantly decreased in the CCI + EA group compared to that in the CCI + SEA group (Fig. 6E). These findings demonstrate that the EA attenuates NP by suppressing ERS in the ACC after CCI.

## Discussion

Here, we demonstrated that both BIP and IRE-1α are expressed in neurons but not in astrocytes or microglia in the ACC. Additionally, we showed that the abundance of BIP and IRE-1α bilaterally increased, while IRE-1α was activated by phosphorylation 7 days after CCI surgery, consistent with the corresponding CCI-induced behavioral hypersensitivity. Intra-ACC microinjection or intraperitoneal injection of the BIP or IRE-1α inhibitor impeded the initiation of CCI-induced NP. Activation of ERS induced hypersensitivity in naive rats. These results indicate that BIP-IRE-1α-mediated ERS in the ACC contributes to peripheral nerve injury-induced NP. Furthermore, EA attenuated hyperalgesia and suppressed BIP overexpression and the activation of IRE-1α during CCI-induced ERS in the ACC, suggesting that EA hampered NP initiation by suppressing ERS in the ACC.

The ACC, a highly heterogeneous cortex with extensive fiber connections, receives and integrates afferent information from other regions of the central nervous system. The ACC has been recognized to play a critical role in the emotional-affective component [23, 24] and has recently emerged as a region involved in modulating the sensory-discriminative component of pain [23]. Indeed, photogenetic studies have demonstrated that specific activation of vertebral neurons in the ACC reduces the threshold of mechanical stimuli in mice [25]. Meanwhile, the activation of inhibitory interneurons significantly inhibits nociceptive perception [26], whereas pharmacological inhibition of chemokine receptor 3 (CXCR3) in the ACC significantly relieves hyperalgesia in NP rats [27]. This evidence suggests that there may be a specific neural circuit involved in nociceptive perception in the ACC, although whether ERS in the ACC is involved in NP needs to be further explored.

Previous studies have shown that ERS plays an important role in the development of neurodegenerative diseases, cerebral ischemia, spinal cord injury, multiple sclerosis, and diabetic neuropathy [28–31]. Furthermore, the BIP-IRE-1α pathway is activated in the peripheral nervous system and spinal cord under NP [10, 32, 33], and local or systemic use of ERS inhibitors could significantly relieve hyperalgesia [34, 35], indicating the important role of BIP-IRE-1α pathway-mediated ERS in NP. However, whether ERS could be triggered in the ACC, and its role in NP have not yet been explored. In the current study, the NP model was established using CCI to investigate the role of ERS in ACC in the sensory-discriminative component of pain. Immunofluorescence analysis revealed that both BIP and IRE-1α were co-localized with NeuN, which was consistent with the findings of a previous study [35, 36], providing the theoretical basis for ERS in the ACC. Next, western blotting was used to detect protein expression to further investigate the role of BIP-IRE-1α-mediated ERS in pain modulation. The results showed bilateral upregulation of BIP and IRE-1α in the ACC after CCI, while IRE-1α was bilaterally activated in a phosphorylated manner, unlike the unilateral activation observed in the spinal cord [35]. This discrepancy may be attributed to the crossover of the spinothalamic tracts in the spinal cord and afferent projections from the contralateral pain-transmitting area [37, 38]. This result indicated the induction of BIP-IRE-1α pathway-mediated ERS after CCI in the ACC. To further explore the role of BIP-IRE-1α-mediated ERS in the ACC during pain modulation, the BIP inhibitor, 4-PBA, and the IRE-1α inhibitor, Kira-6, were consecutively administered via ACC microinjection and intraperitoneal injection respectively to inhibit CCI-induced ERS. Behavioral tests revealed that both 4-PBA and Kira-6 impeded the initiation of mechanical hyperalgesia and cold allodynia after CCI, suggesting a critical role for ERS in the ACC in CCI-induced NP. Interestingly, there were no significant behavioral differences among the four groups on day 3 after CCI, but significant differences were observed on days 5 and 7. This phenomenon may be attributed to the delayed action of the drugs, or incision-mediated nociceptive hyperactivity that was not modulated by ERS in the ACC. However, the exact mechanism underlying this phenomenon requires further investigation. In line with the results of previous studies [34], the results of western blotting showed that both 4-PBA and Kira-6 significantly suppressed the expression of IRE-1α and its phosphorylation, indicating successful inhibition of ERS in the ACC.

JNK and p38 are well-recognized kinases involved in pain modulation. We analyzed pJNK and pP38 expression using western blotting, with the results showing a significant decrease in expression within the ACC of rats administered Kira-6. This reduction implies a potential connection between the inhibition of ERS and the downregulation of P38 and JNK, which may contribute to reduced production of inflammatory cytokines.

The suppression of ERS has potential side effects, including disruptions in the intricate process of protein folding, disturbances in the regulation of intracellular calcium, and modifications to the unfolded protein response pathway [39]. In the present study, we evaluated locomotor ability using forelimb grip strength, but found no significant differences in limb strength between the rats treated with inhibitors and the control group, suggesting that the application of ER inhibitors does not impair the locomotive ability of rats. However, given the limitations inherent to animal studies, other side effects, such as abnormal sensations, could not be ruled out.

**Fig. 7:**
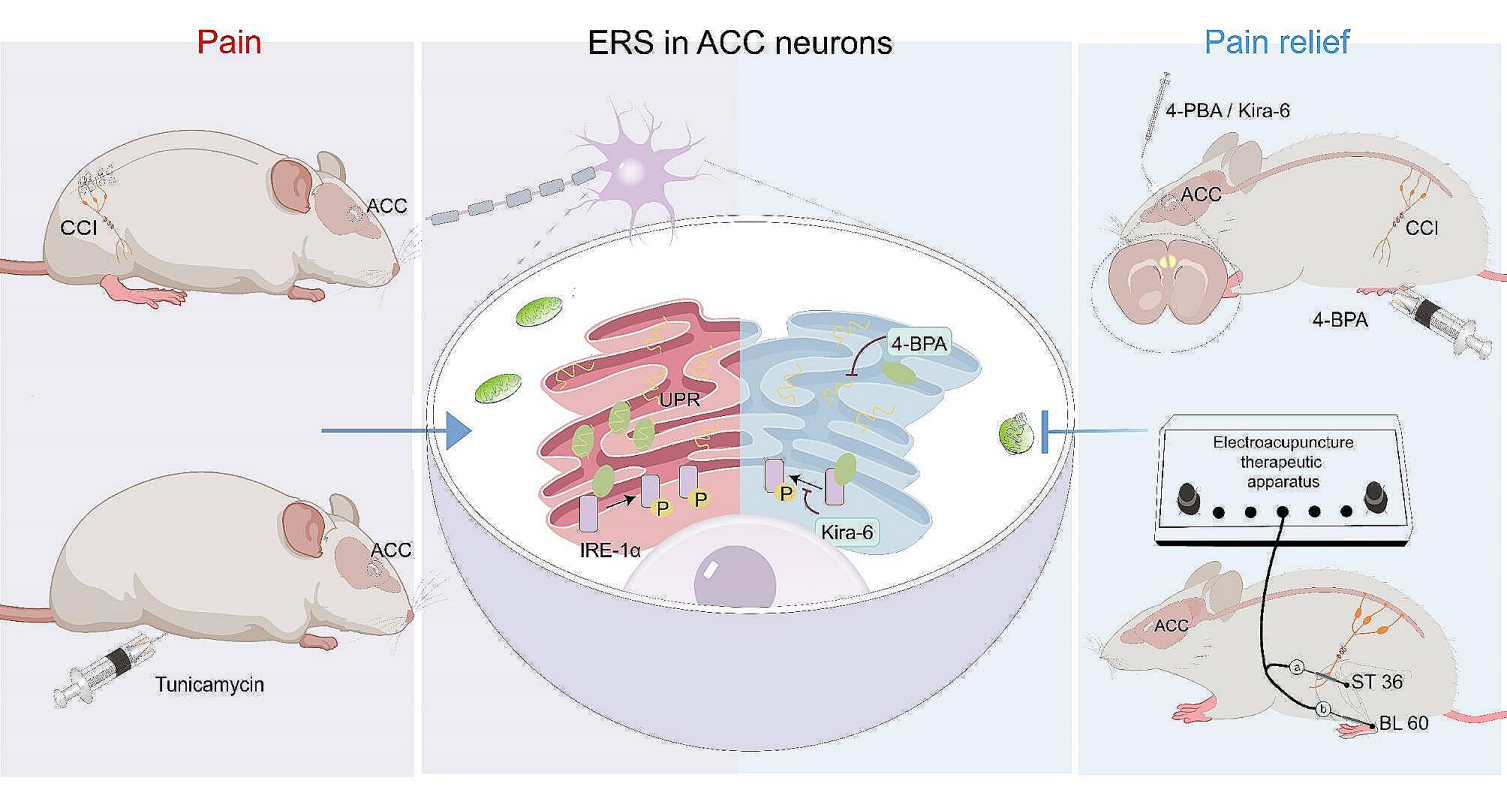
An explanatory graphic illustrating electroacupuncture attenuates neuropathic pain by suppressing endoplasmic reticulum stress in the anterior cingulate cortex. ERS: Endoplasmic reticulum stress, ACC: Anterior cingulate cortex, UPR: Unfolded protein response

Previous studies have confirmed that the mechanisms underlying the analgesic effect of EA are via the regulation of opioids, norepinephrine, or cholinergic activity in subcortical structures such as the spinal cord, periaqueductal gray matter, and hypothalamus pre-optic area [16, 40–42]. However, whether the cerebral cortex is involved in the antihyperalgesic effects of EA remains unclear. By regulating ERS, EA can alleviate neuronal apoptosis caused by ischemic stroke or spinal cord injury and promote the recovery of nerve function [43, 44], providing a theoretical possibility for EA to alleviate NP by suppressing ERS. In the present study, we performed EA treatment from days 0 to 6 for 30 min/day. The behavioral results showed no significant differences between the baseline and day 3 after CCI; however, the MWT was significantly increased and the ATS was significantly decreased in the CCI-EA group on days 5 and 7 after CCI, indicating that consecutive EA treatment effectively hampered the initiation of NP after CCI. Similarly, the delayed antihyperalgesic effect of EA might be attributed to the delayed action of the EA, or incision-induced nociceptive hyperactivity and acute nervous inflammation were not modulated by ERS in the ACC. Western blotting was used to further elucidate the mechanisms underlying the antihyperglycemic effect of EA, with the results showing that EA inhibited the overexpression of BIP and the CCI-induced activation of IRE-1α, suggesting that EA attenuated NP by suppressing BIP-IRE-1α-mediated ERS in the ACC (Fig. 7).

### Limitations

Our study has limitations that warrant discussion. First, although we have revealed that the BIP-IRE-1α pathway in the ACC was activated after peripheral nerve injury, that both the antagonists of BIP and IRE-1α mitigated CCI-induced NP, and that the activation of this pathway induces pain hypersensitivity, the mechanisms underlying ERS mediating NP were not well-addressed. Second, only one dose of inhibitors was tested in the present study, and we do not know whether the effect is dose-dependent or not. Third, studies demonstrated that IRE-1α-, ATF6-, and PERK-mediated ERS played an important role in the development of neuropathic pain in the spinal cord [35], but the role of ATF6 and PERK in the ACC during NP and whether they are involved in the high central mechanisms of the antihyperalgesic effects of EA need to be further elucidated.

## Conclusions

The present study demonstrated that BIP-IRE-1α-mediated ERS in the ACC plays a critical role in the initiation of NP and that EA suppresses the initiation of NP induced by peripheral nerve injury by suppressing BIP-IRE-1α-mediated ERS in the ACC. We present novel evidence that ERS in the ACC is implicated in the development of NP and provide insight into the molecular mechanisms involved in the analgesic effect of EA.

## Materials and methods

### Animals

**Fig. 8:**
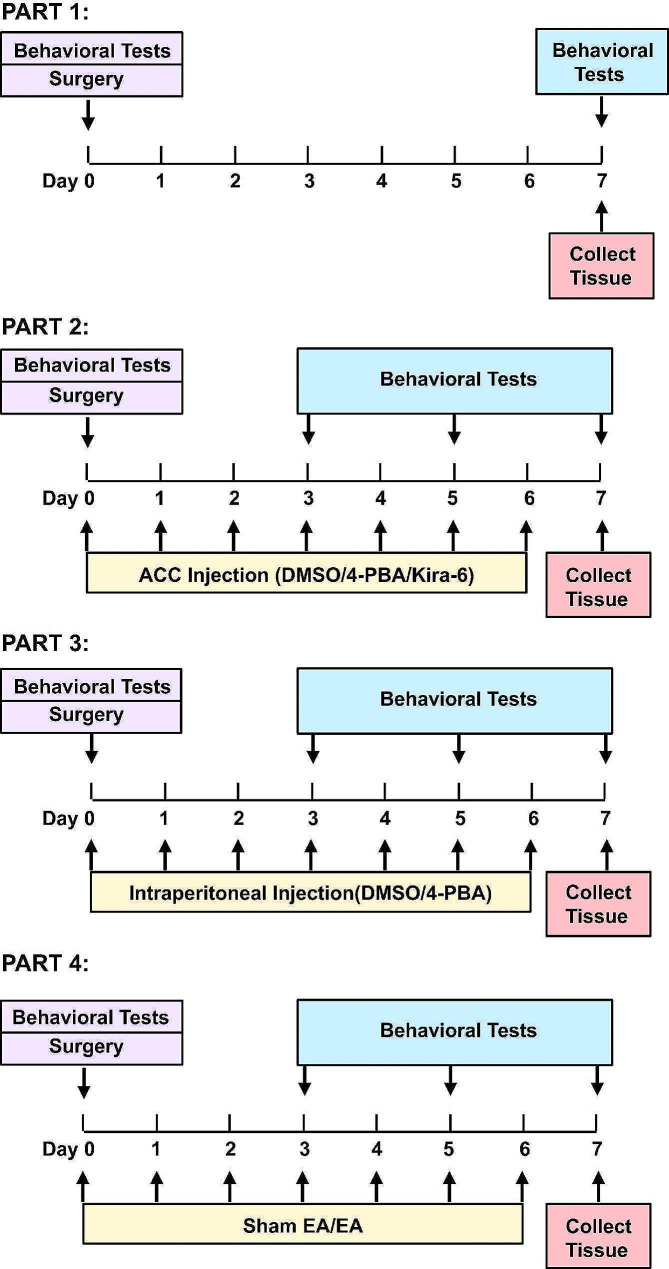
Illustration of the experimental timelines

Male Sprague–Dawley rats (6–8 weeks old; weight, 220 ± 20 g) were obtained from the Experimental Animal Center of the Zhejiang Academy of Medical Sciences. Rats were housed in groups (3–4 per cage) at a temperature of 24 ± 2 °C under a 12-h light/dark cycle, with food and water provided *ad libitum*. The rats were randomly allocated to each group and allowed a week to adapt to the new environment before initiating the experiment. All animal experiments complied with the ARRIVE guidelines, internationally accredited guidelines, and ethical regulations on animal research [45]. The study protocol was approved by the Research Ethics Committee of the First Affiliated Hospital of Zhejiang University. All efforts were made to minimize the number of animals used and their suffering. The experimental timeline used in this study is shown in Fig. 8.

### Induction of NP

After baseline had been obtained, the rats were randomly assigned to the sham and chronic constriction injury (CCI) groups. Under isoflurane anesthesia, as reported by Bennett and Xie and in our previous studies [46–48], the left sciatic nerve of the rat was exposed through blunt dissection of the middle thigh. In the CCI group, the sciatic nerve was isolated and ligated using three strands of 4 − 0 chromic gut sutures (Pudong Jinhuan Co. Ltd., Shanghai, China) placed 1 mm apart. The muscles and skin were closed layer-by-layer with 4 − 0 sutures. In the sham group, the left sciatic nerve was visualized but not ligated. After surgery, the rats were subcutaneously injected with 80,000 U of penicillin to prevent infection.

### Behavioral tests

The rats were allowed to acclimate for 3 consecutive days (30 min per day) in a plastic box (12 cm × 15 cm × 22 cm) on an elevated wire mesh before behavioral tests. The experimenter was blinded to the treatment received by the rats.

### Mechanical withdrawal threshold

The mechanical withdrawal threshold (MWT) was determined using a set of von Frey filaments. Briefly, the left plantar surface was stimulated with filaments of increasing stiffness (0.4–26 g) until a quick withdrawal or licking of the paw was noted, and the magnitude of the filaments was recorded as the MWT. The testing was repeated three times with an interval of 5 min, and the average value was considered the final MWT.

### Acetone test score

Cold allodynia was tested using the acetone test score (ATS), as described by Farsi et al. [49]. on the same apparatus as the MWT test. Briefly, 100 µL of acetone was sprayed onto the left plantar surface, and the responses were observed for 20 s after application. The results were scored on a 4-point scale as follows: 0, no response; 1, startle response without paw withdrawal; 2, brief withdrawal of the paw; 3, prolonged withdrawal (5–30 s); and 4, prolonged and repetitive withdrawal along with flinching and/or licking. The testing was repeated three times at 5-min intervals, and the average value was considered as the final ATS.

### ACC catheterization and drug administration

For intra-ACC drug administration, ACC catheterization was performed under anesthesia with pentobarbital sodium (60 mg/kg) according to our previous report [27]. Briefly, the head was fixed on a stereotactic apparatus in the prone position, and a 1-cm longitudinal incision was made in the middle of the head to expose the skull. Two holes were drilled on each side (Bregma forward, 1.7 mm; lateral, 0.6 mm) and a trocar (Shenzhen Ruiwode Life Science and Technology Co., Ltd, Guangdong, China) was inserted. Two small screws were installed superficially in the occipital bone, dental methyl methacrylate was used to fix the trocar with screws, and the rats were administered a subcutaneous injection of 80,000 U penicillin to prevent infection. CCI was performed 7 days after catheterization. The BIP and IRE-1 inhibitors 4-PBA and Kira-6 were purchased from Selleck (Houston, TX, USA) and dissolved in 10% dimethyl sulfoxide (DMSO). The rats were randomly assigned to the following four groups: CCI, CCI + DMSO, CCI + 4-PBA, and CCI + Kira-6. Both 4-PBA and Kira-6 were dissolved in 10% DMSO at a concentration of 20 µg/µL. Drugs or vehicle were bilaterally injected into the ACC (1 µL per side) from days 0 to 6 after CCI surgery.

### Systemic administration

For systemic administration, intraperitoneal injections of 4-PBA (dissolved in 10% DMSO) were administered on the day of CCI and continued once a day for the following 7 days at a dosage of 20 mg/kg.

For ERS activation, a single intraperitoneal injection of tunicamycin, a recognized activator of ERS, was administered to naive rats. Tunicamycin was obtained from Aladdin Reagent (Shanghai, China) and dissolved in 10% DMSO. The rats were randomly assigned to the naive, DMSO (10% DMSO, 1 mL), and tunicamycin (2 µg/kg, in a 1 mL volume) groups. All animals underwent behavioral testing before and 2 and 24 h after administration.

### Locomotive ability

We used the YLS-13 A grasp tester (Jinan Yiyan Science Co. Ltd., Shandong, China) to measure the grip force of the anterior claw and evaluate locomotive ability. The experimental protocol involved horizontally situating the grip tester on the ground, placing the rat onto a flat plate, and firmly fastening the front paw onto the steel wire. The incremental force was gradually applied against gravity until the front paw released its grip on the wire. Subsequently, the grip force was automatically recorded. During each session, three measurements were collected at 5-min intervals, and the average was computed to determine the final grip force.

### Electroacupuncture

The EA procedure was performed according to the methods outlined in a previous study [50]. After baseline was obtained, the rats were randomly allocated to the following three groups: CCI, CCI + sham EA (CCI + SEA), and CCI + EA (CCI + EA). Briefly, acupuncture needles (diameter, 0.25 mm; depth, 4 mm) were inserted into the left Zusanli (ST36, 5 mm lateral to the anterior tubercle of the tibia) and left Kunlun (BL60; the sunken area between the lateral malleolus and the Achilles tendon) acupoints. The needles were connected to a HuaTuo acupuncture nerve stimulator (HuaTuo-SDZ-II; Suzhou Medical Appliance Co., Ltd., Suzhou, Jiangsu). The EA parameters were set as follows: 2 Hz, consecutive wave output, which lasted for 30 min, with intensities ranging from 0.5 to 1.5 mA (increased by 0.5 mA every 10 min). For sham EA treatment, needles were inserted as in the EA group, but without electrical stimulation. EA treatment was performed once daily for 7 consecutive days, from days 0 to 6 after CCI.

### Immunofluorescence assay

After deep anesthesia with pentobarbital sodium, the rats were transcardially perfused with 150 mL of 1 × phosphate buffered saline (PBS) (4 °C), followed by 150 mL of 4% paraformaldehyde (4 °C). The ACC was harvested, fixed with 4% paraformaldehyde for 48 h, and then dehydrated with 30% sucrose for 3 days at 4 °C. Subsequently, the ACC was transversely cut into slices (30-µm thick). The sections were blocked with 10% sheep or donkey serum for 2 h at room temperature and incubated with the following primary antibodies for 48 h at 4 °C: goat-anti-Iba1 (1:200, Abcam, Cambridge, UK), mouse anti-GFAP (1:500, Cell Signaling Technology), mouse anti-NeuN (1:2000, Abcam), rabbit anti-BIP (1:500, Cell Signaling Technology), and rabbit-anti-IRE-1 (1:1000, Proteintech). The sections were washed with 1 × PBS and incubated with fluorescent secondary antibodies in the dark for 2 h at room temperature. Finally, the sections were examined under a fluorescence microscope (FV3000; Olympus, Tokyo, Japan).

### Western blot analysis

After deep anesthesia with pentobarbital sodium, the rats were decapitated, the brain was harvested, and the ACC was divided into the left (Ipsi.) and right (Contra.) sections as described in our previous study [27]. The ipsilateral protein samples were separated by SDS-PAGE and transferred to a PVDF membrane. Subsequently, the membranes were blocked in 5% skim milk at room temperature for 1 h before incubation with the following primary antibodies for 24 h at 4 °C: rabbit-anti-BIP (1:500, Cell Signaling Technology, Danvers, MA, USA), rabbit-anti-IRE-1 (1:1000, Proteintech, Rosemont, IL, USA), rabbit-anti-pIRE-1 (1:1000, Proteintech), and mouse-anti-GAPDH (1:10,000, Proteintech). After washing with TBST, the membrane was incubated with an HRP-conjugated secondary antibody for 2 h at room temperature. The ChemiDoc MP System (Bio-Rad, Hercules, CA, USA) was used to detect complex immune bands.

### Statistical analysis

All data are expressed as the mean ± SEM and were analyzed using GraphPad Prism 8.0 (GraphPad, San Diego, CA, USA). Behavioral data were compared between the two groups using independent *t*-tests. The results of behavioral tests across different time points were analyzed using repeated measures two-way analysis of variance (ANOVA), followed by Bonferroni’s post hoc test. Western blot data were analyzed using one-way ANOVA, followed by Tukey’s multiple comparisons test. Statistical significance was set at *p* < 0.05.

### Electronic supplementary material

Below is the link to the electronic supplementary material.

Supplementary Material 1

## Data Availability

The datasets generated during and/or analyzed during the study are available from the corresponding author on reasonable request.

## Abbreviations

NP: Neuropathic pain
ERS: Endoplasmic reticulum stress
EA: Electroacupuncture
ACC: Anterior cingulate cortex
CCI: Chronic constriction injury
BIP: Glucose-regulated protein 78
IRE-1α: Inositol-requiring enzyme 1α
MWT: Mechanical withdrawal threshold
ATS: Acetone test score
ANOVA: Analysis of variance
NeuN: Neuron-specific nuclear protein
GFAP: Glial fibrillary acidic protein
Iba1: Ionized calcium-binding adaptor molecule-1
