# Supplementary material for: Electroacupuncture attenuates neuropathic pain via suppressing BIP-IRE-1α-mediated endoplasmic reticulum stress in the anterior cingulate cortex
Source: Biol Res. 2024 May 29;57:34. doi: 10.1186/s40659-024-00511-3 (PMC11134655; doi:10.1186/s40659-024-00511-3)
Supplement: Supplementary file 1 — Supplementary Material 1 [file 40659_2024_511_MOESM1_ESM.docx]

**Electroacupuncture attenuates neuropathic pain via suppressing BIP-IRE-1α-mediated endoplasmic reticulum stress in the anterior cingulate cortex**

Lin-Wei Ma, Yu-Fan Liu, Hui Zhang, Chang-Jun Huang, Ang Li, Xin-Zhe Qu, Jia-Piao Lin, Yan Yang, Yong-Xing Yao

**List of Supplementary Information**

**Original blot images for statistics**

Fig S3. Original blots for Figure 3C.

Fig S3. Original blots for Figure 3D.

Fig S3. Original blots for Figure 3E.

Fig S4. Original blots for Figure 4F.

Fig S4. Original blots for Figure 4G.

Fig S4. Original blots for Figure 4H.

Fig S4. Original blots for Figure 4I.

Fig S4. Original blots for Figure 4J.

Fig S5. Original blots for Figure 5D.

Fig S5. Original blots for Figure 5E.

Fig S5. Original blots for Figure 5F.

Fig S7. Original blots for Figure 7C.

Fig S7. Original blots for Figure 7D.

Fig S7. Original blots for Figure 7E.


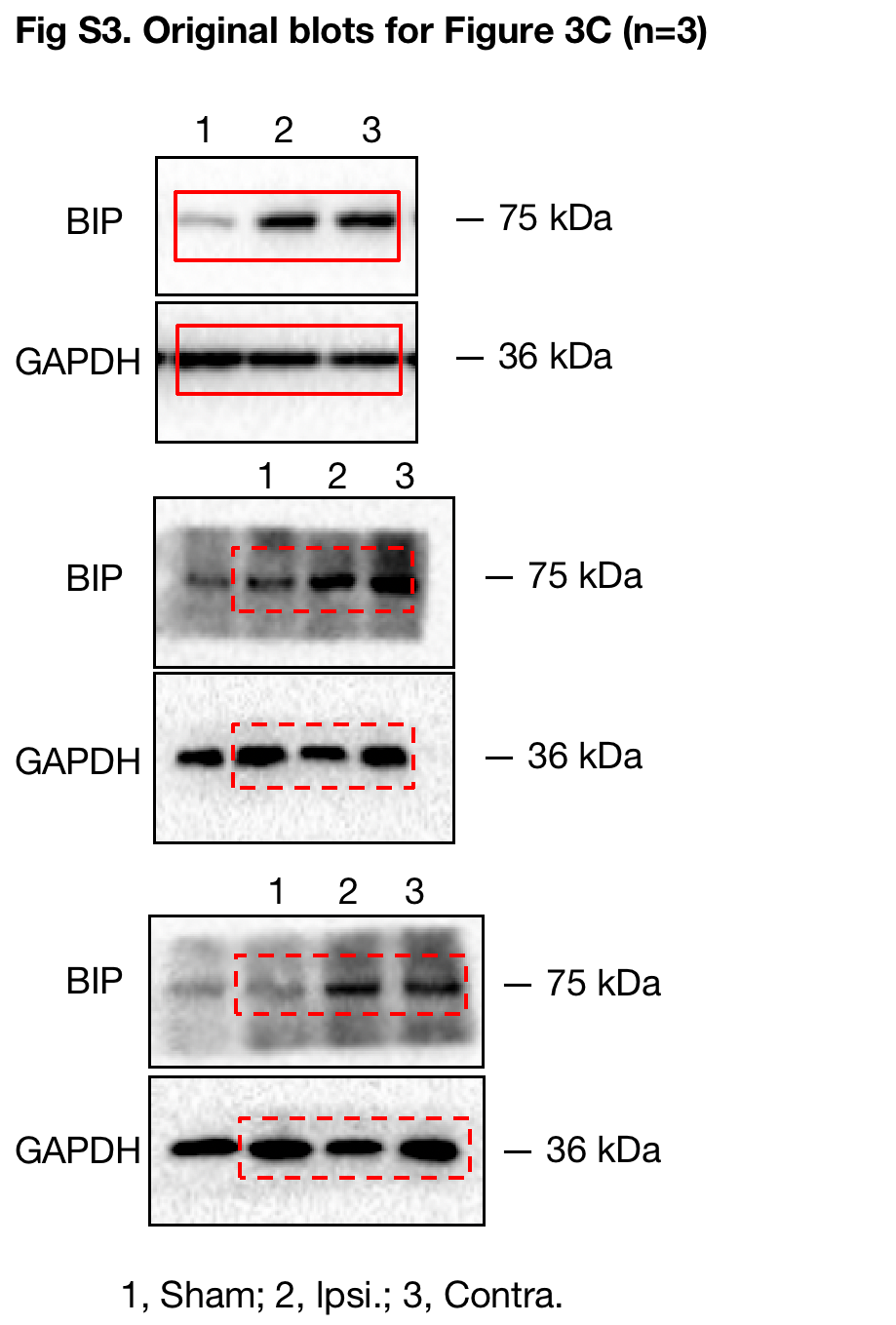


**
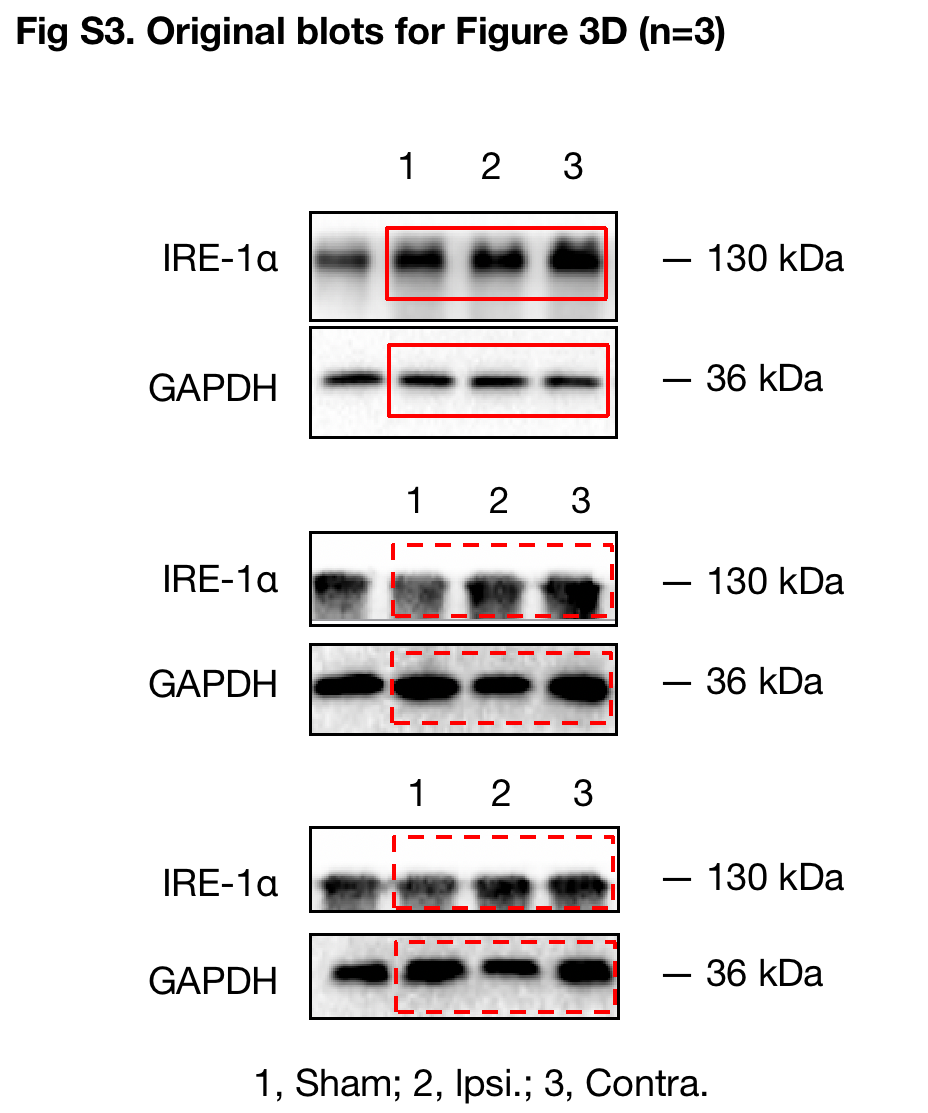
**

**
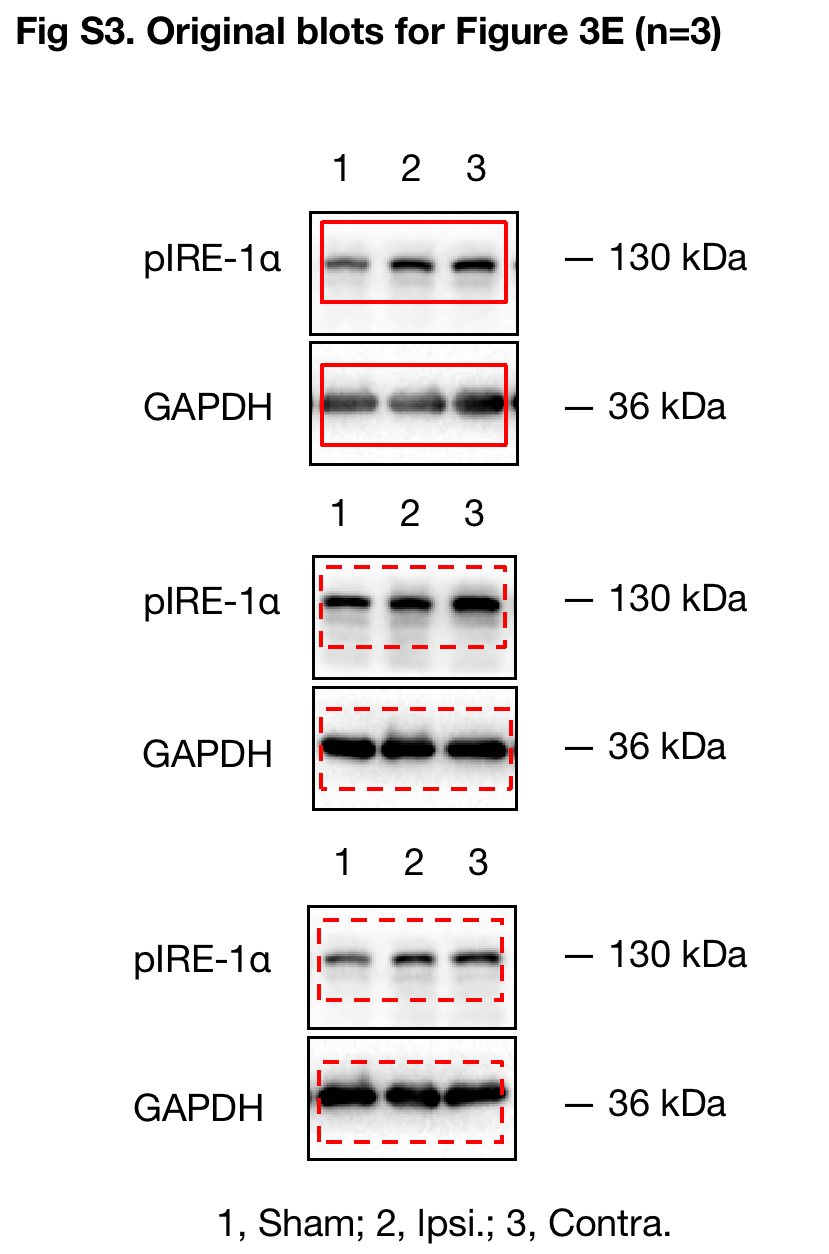
**

**Fig S4. Original blots for Figure 4F (n=3)**

**
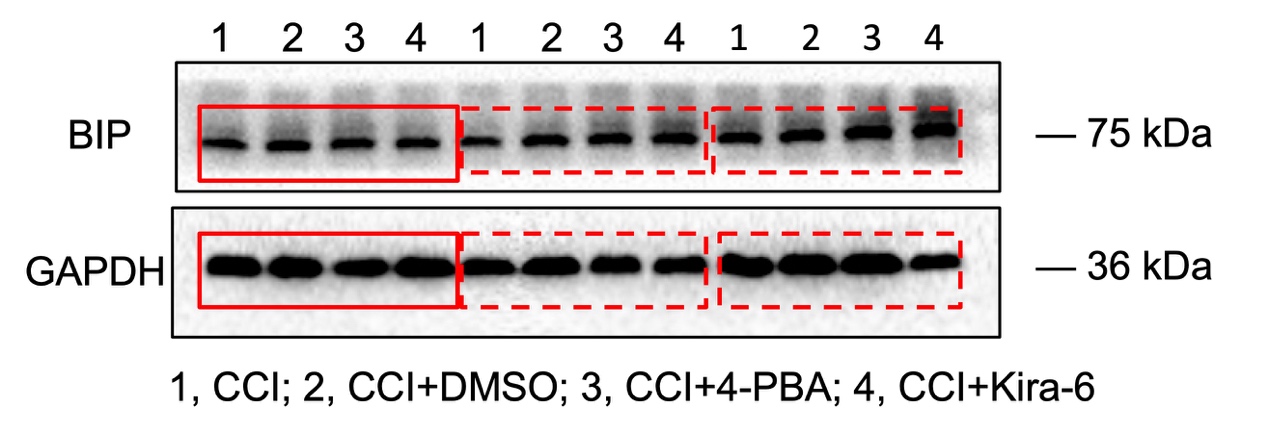
**

**Fig S4. Original blots for Figure 4G (n=3)**

**
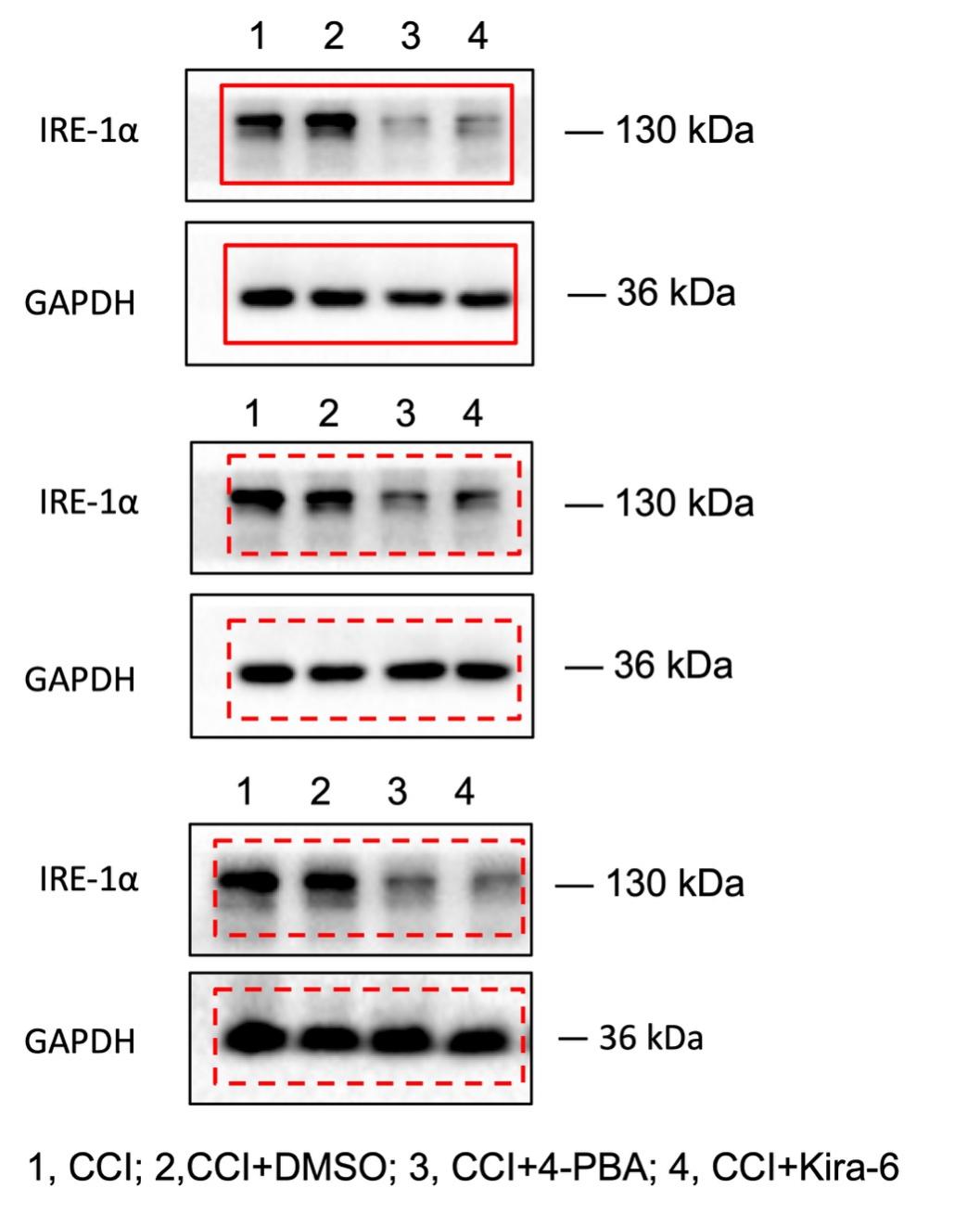
**

**Fig S4. Original blots for Figure 4H (n=3)**

**
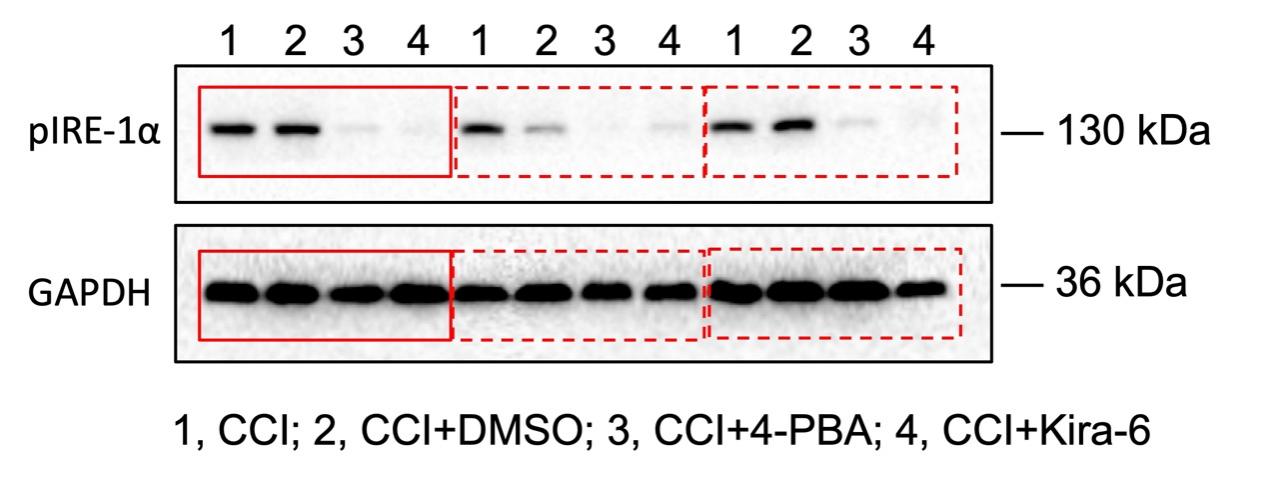
**

**
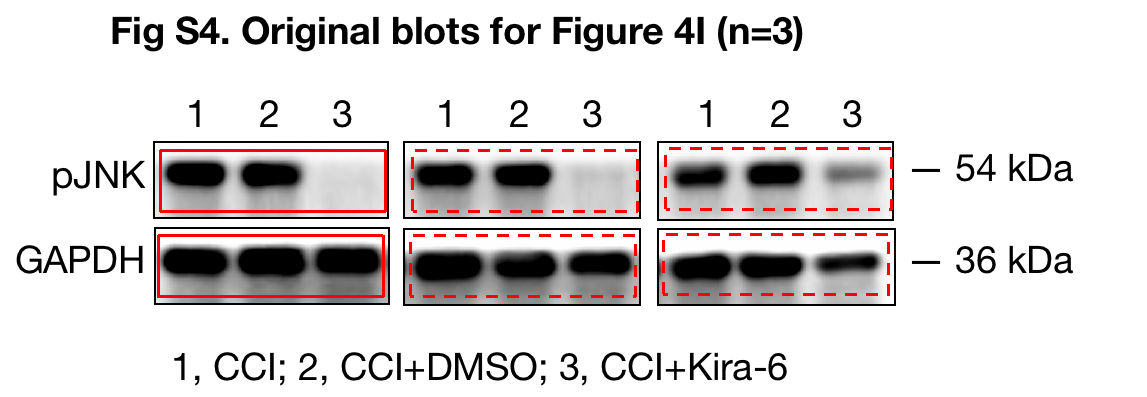
**

**
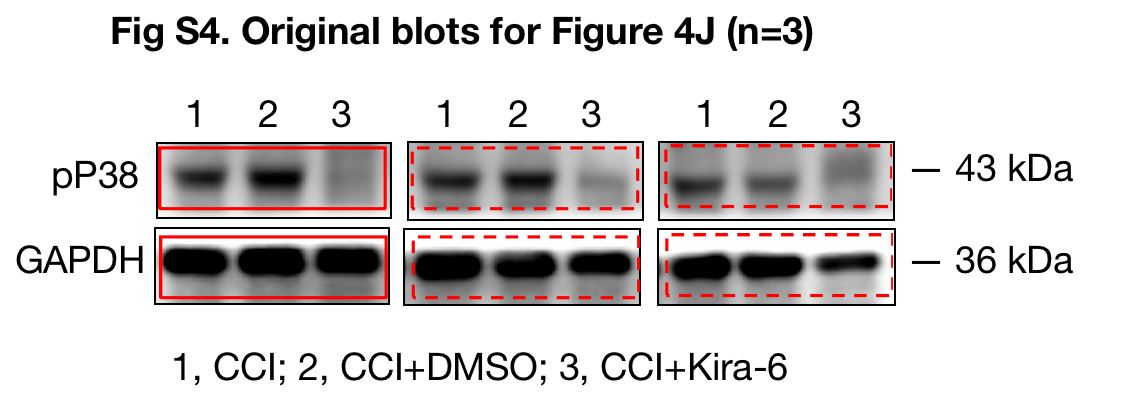
**

**
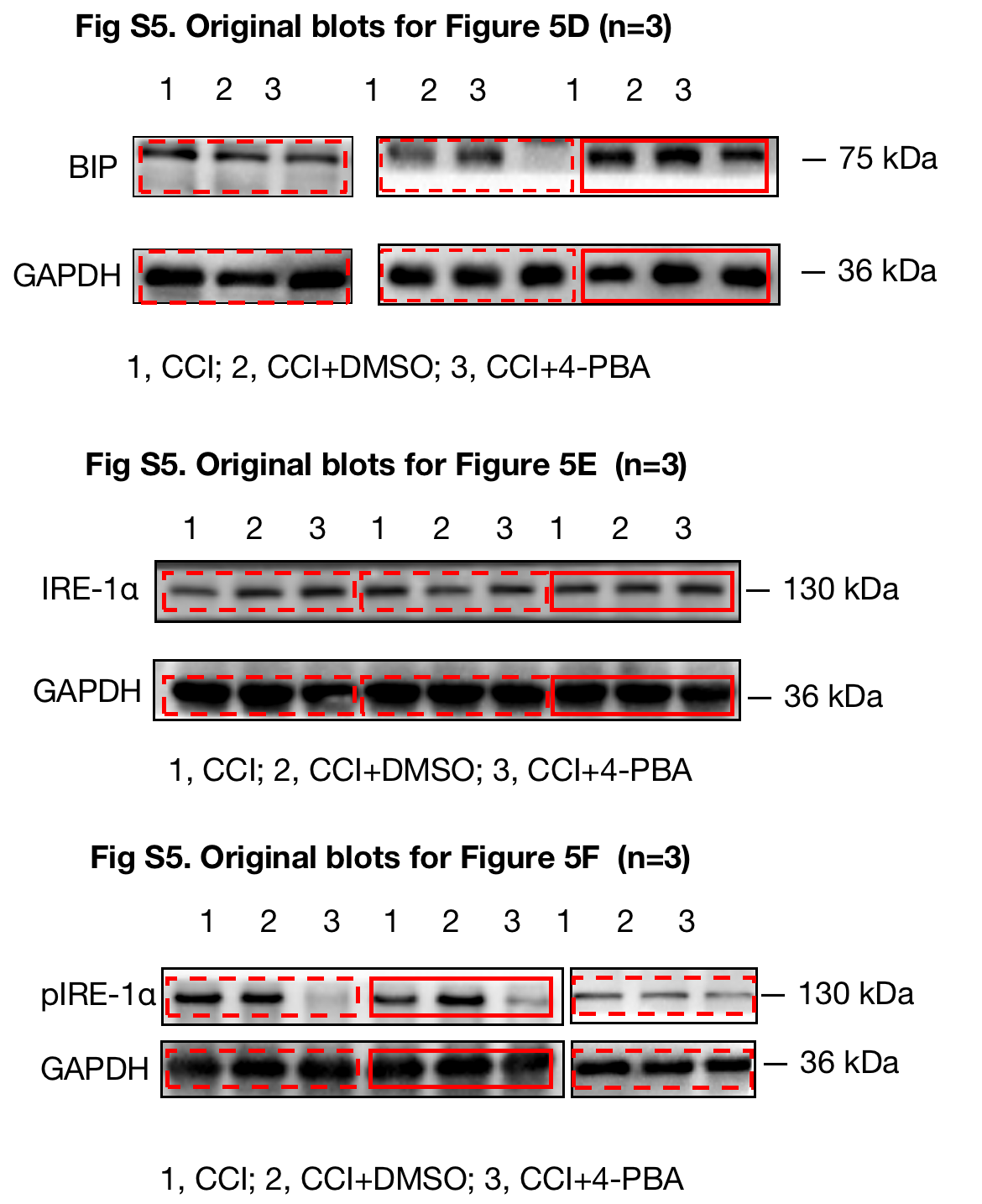
**

**
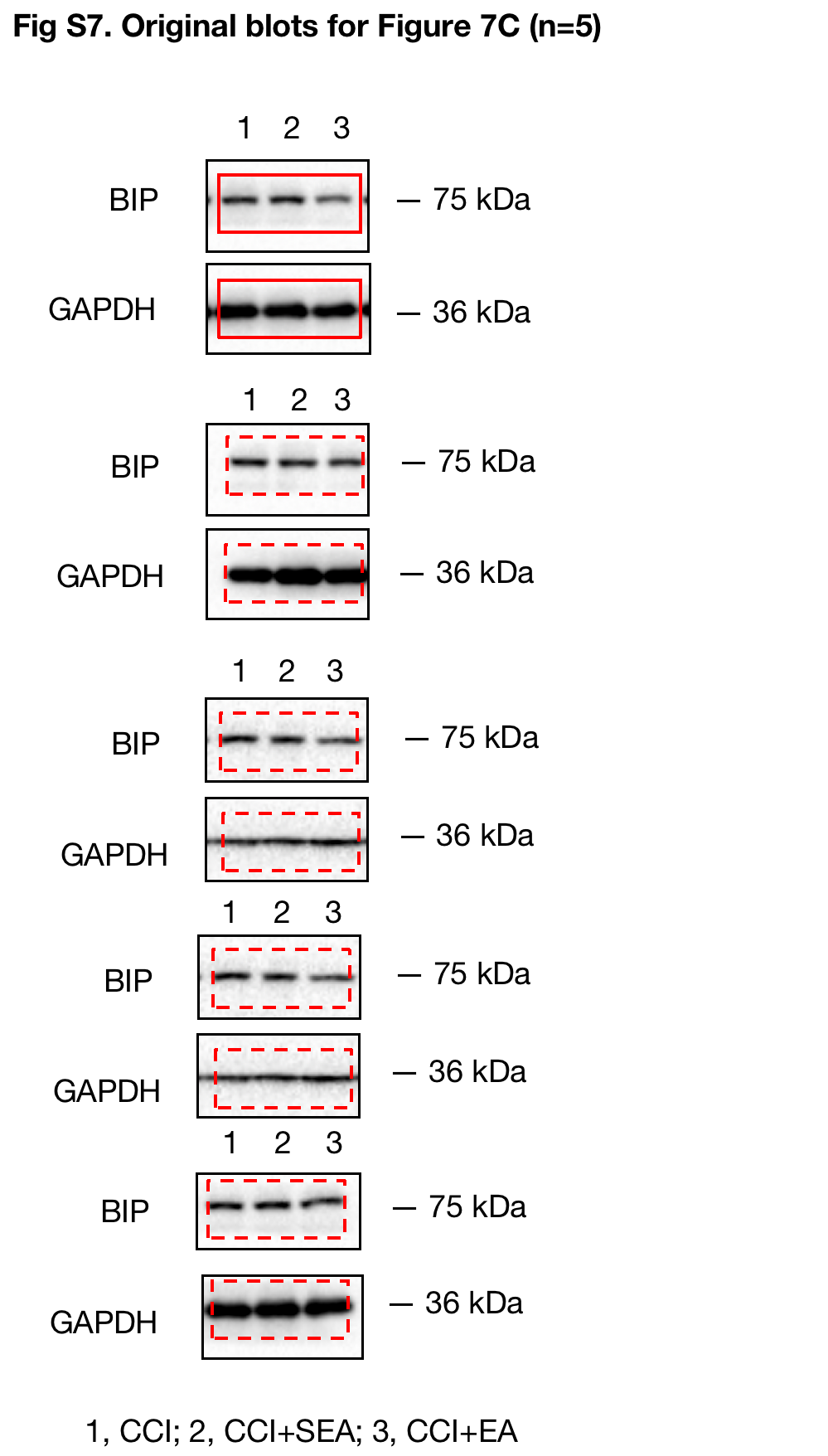
**

**
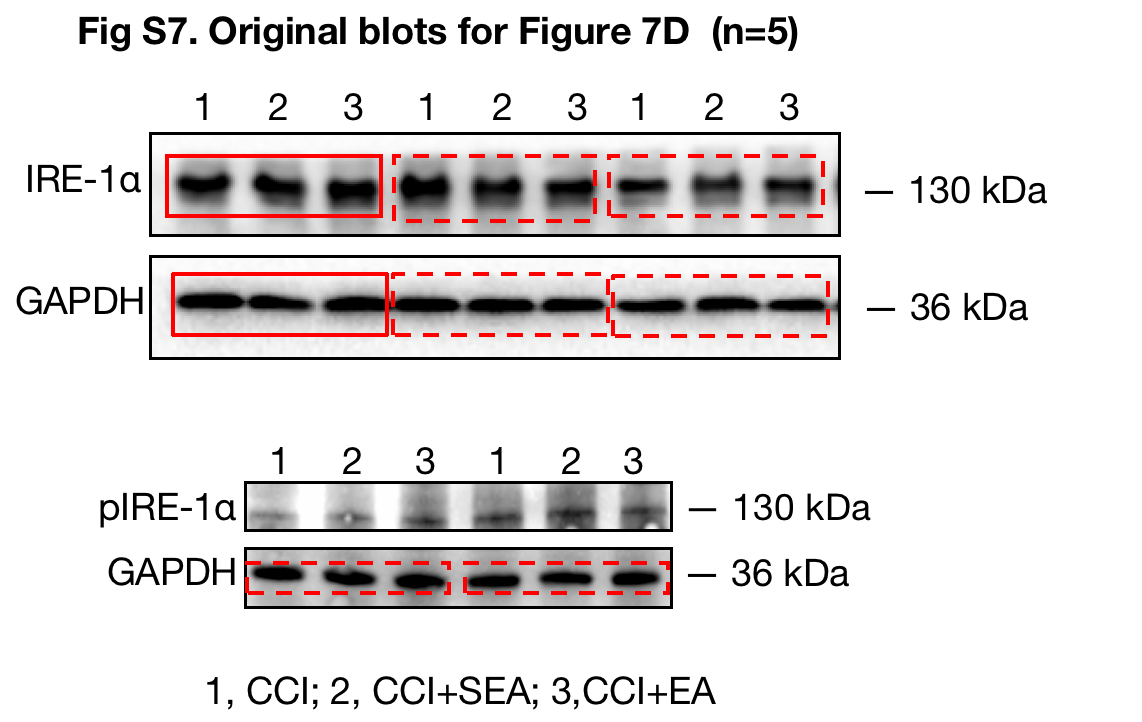
**

**
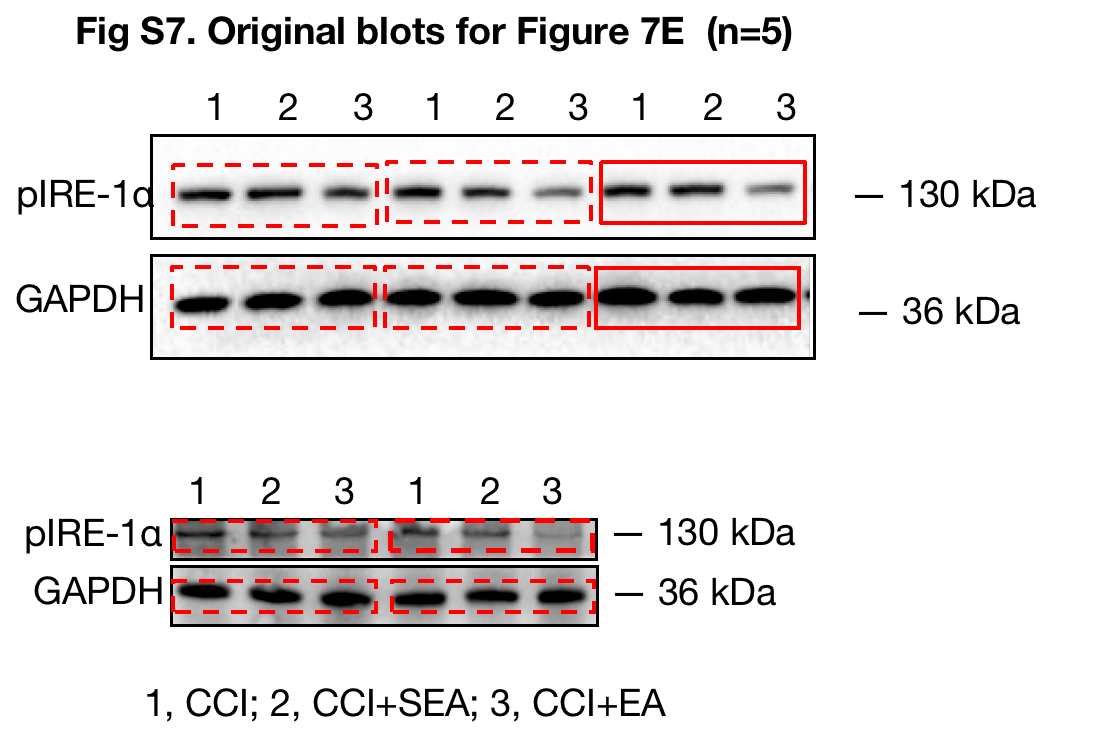
**
